# Synthesis of Pseudellone Analogs and Characterization as Novel T-type Calcium Channel Blockers

**DOI:** 10.3390/md16120475

**Published:** 2018-11-28

**Authors:** Dan Wang, Pratik Neupane, Lotten Ragnarsson, Robert J. Capon, Richard J. Lewis

**Affiliations:** Division of Chemistry and Structural Biology, Institute for Molecular Bioscience, the University of Queensland, Brisbane, Qld 4072, Australia; dan.wang@imb.uq.edu.au (D.W.); p.neupane@imb.uq.edu.au (P.N.); l.ragnarsson@imb.uq.edu.au (L.R.)

**Keywords:** Ca_V_3.x blockers, bisindole alkaloid, marine fungal product, pseudellone C

## Abstract

T-type calcium channel (Ca_V_3.x) blockers are receiving increasing attention as potential therapeutics for the treatment of pathophysiological disorders and diseases, including absence epilepsy, Parkinson’s disease (PD), hypertension, cardiovascular diseases, cancers, and pain. However, few clinically approved Ca_V_3.x blockers are available, and selective pharmacological tools are needed to further unravel the roles of individual Ca_V_3.x subtypes. In this work, through an efficient synthetic route to the marine fungal product pseudellone C, we obtained bisindole alkaloid analogs of pseudellone C with a modified tryptophan moiety and identified two Ca_V_3.2 (**2**, IC_50_ = 18.24 µM; **3**, IC_50_ = 6.59 µM) and Ca_V_3.3 (**2**, IC_50_ = 7.71 µM; **3**, IC_50_ = 3.81 µM) selective blockers using a FLIPR cell-based assay measuring Ca_V_3.x window currents. Further characterization by whole-cell patch-clamp revealed a preferential block of Ca_V_3.1 activated current (**2**, IC_50_ = 5.60 µM; **3**, IC_50_ = 9.91 µM), suggesting their state-dependent block is subtype specific.

## 1. Introduction

Alkaloids produced by marine animals are mostly potent cytotoxins evolved for defense [1]. Marine indole alkaloids have been widely explored for their therapeutic potentials, providing potential new drug leads for the treatment of a wide range of diseases including cancer, neurological disorders, and parasitic infections [2,3]. However, the underlying pharmacological targets and mechanisms of the bioactive indole alkaloids remain largely undefined. Triptans, a family of tryptamine-based drugs used for the abortive treatment of migraine headaches, have been well established and characterized as selective agonists of 5-HT_1 B_ and 5-HT_1 D_ serotonin receptors [4]. As a result, pharmacological studies on indole alkaloids mainly focus on the discovery of novel serotonin receptor agonists [5]. Compounds with an indole moiety have been explored for their activities on voltage-gated ion channels previously, and a series of tremorgenic indole alkaloids have been reported to potently block potassium channels in smooth muscle [6]. In a more recent study, a novel aryl indole compound was identified as a potent and selective blocker of N-type Ca_V_2.2, and showed robust in vivo efficacy in inflammatory and neuropathic rat pain models [7].

In 2015, Liu et al. isolated a tryptophan-derived bisindole alkaloid pseudellone C (**1**) from the marine fungus *Pseudallescheria ellipsoidea* along with two other epipolythiopiperazine alkaloids [8]. Following this discovery, in 2017 Sathieshkumar et al. reported the total synthesis of pseudellone C (44% overall yield) [9], although the biological activity of these natural products remains unstudied. Here, we describe an efficient total synthesis of pseudellone C and several new bisindole alkaloid analogs. To investigate their pharmacological potential, we explored their activity in voltage-gated calcium channels (VGCCs) using FLIPR cell-based assays, and further characterized two potent low voltage-activated (LVA) T-type calcium channel (Ca_V_3.x) blockers by whole-cell patch-clamp using an automated electrophysiology platform, QPatch 16 X. For the first time, we identified two “pseudellone” bisindole alkaloids as potent and selective Ca_V_3.x blockers, demonstrating their potential as leads for the development of new analgesic and antiepileptic agents.

## 2. Results

### 2.1. Synthesis of Pseudellone C and Its Bisindole Alkaloid Analogs

**Scheme 1:**
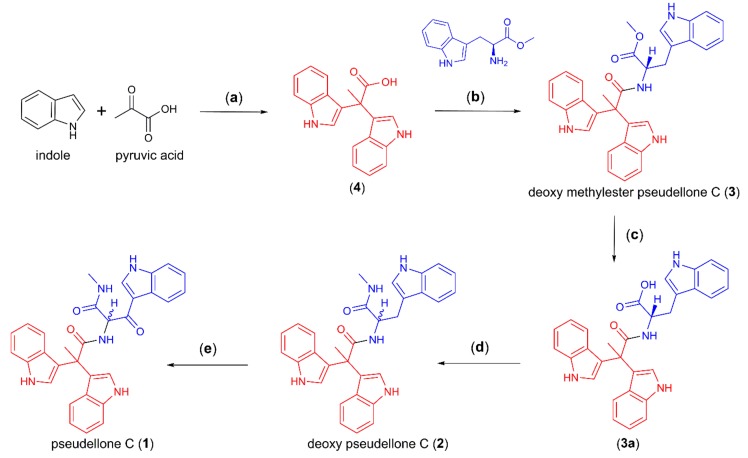
Synthesis of pseudellone C (**1**) and analogs **2**, **3**, **3a,** and **4**. (**a**) MeCN, FeCl_3_, r.t., 12 h, 85%; (**b**) DCC, benzotriazole, TEA, DMF, 60 °C, 1 h, 38%; (**c**) LiOH, MeOH/H_2_O (1:1), r.t., 5 h, 85%; (**d**) DCC, methylamine, benzotriazole, DMF, 60 °C, 1 h, 37%; (**e**) DDQ, MeOH/H_2_O (1:1), 50%.

The synthesis of 2,2-bis (3,3′-indolyl) propionic acid (**4**) (85% yield) was achieved by a Friedel–Crafts condensation of indole and pyruvic acid [10]. As tryptophan is well known as a precursor for a wide range of pharmacologically important indole alkaloids [11,12], we employed a one-pot synthesis of deoxy-pseudellone C (**2**) by first coupling **4** with tryptophan methyl ester to yield **3** (38%) [9], followed by LiOH-mediated hydrolysis to yield the free acid **3a** (85% yield), then DCC-initiated amide coupling of **3a** with methylamine to yield deoxy-pseudellone C (**3a**) (37% yield, albeit with racemization about the single chiral center), and finally, DDQ oxidation of **2** to yield pseudellone C (**1**) (50% yield) (Scheme 1).

### 2.2. Evaluation of VGCC Activities of the Synthetic Compounds Using FLIPR Cell-Based Assays

Pseudellone C (**1**), along with its bisindole alkaloid analogs **2**, **3**, **3a**, and its bisindole substrate **4,** were evaluated for activity on VGCCs using FLIPR cell-based assays (see Table 1).

**Figure 1:**
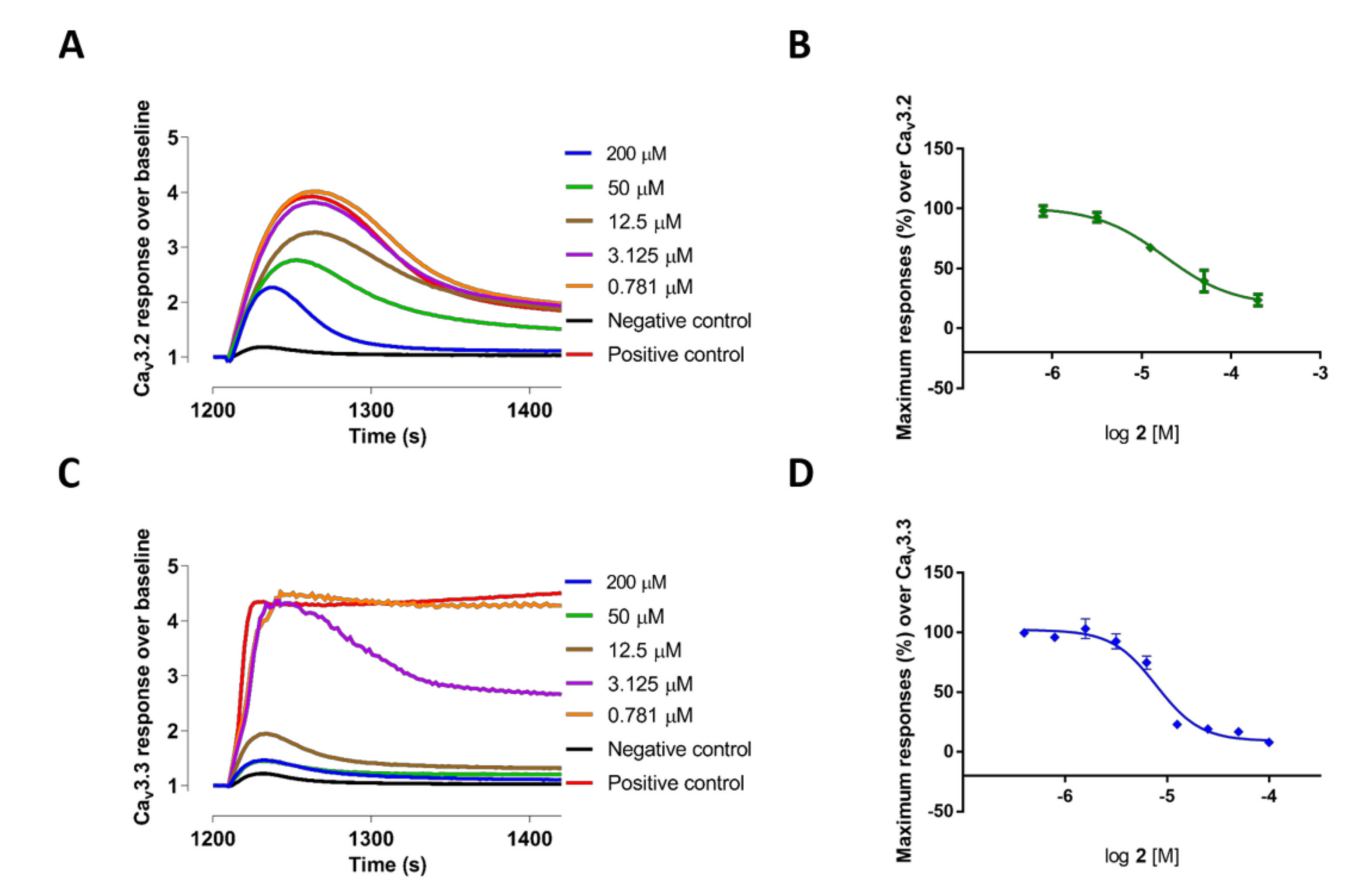
(**A**) Representative fluorescent traces of the Ca_V_3.2 window current before and after addition of compound **2**; (**B**) the concentration-response curve for compound **2** (IC_50_ = 18.16 μM, *n =* 4); (**C**) representative fluorescent traces of the Ca_V_3.3 window current before and after addition of compound **2**; and (**D**) the concentration response curve for compound **2** (IC_50_ value of 7.83 μM, *n =* 4).

**Figure 2:**
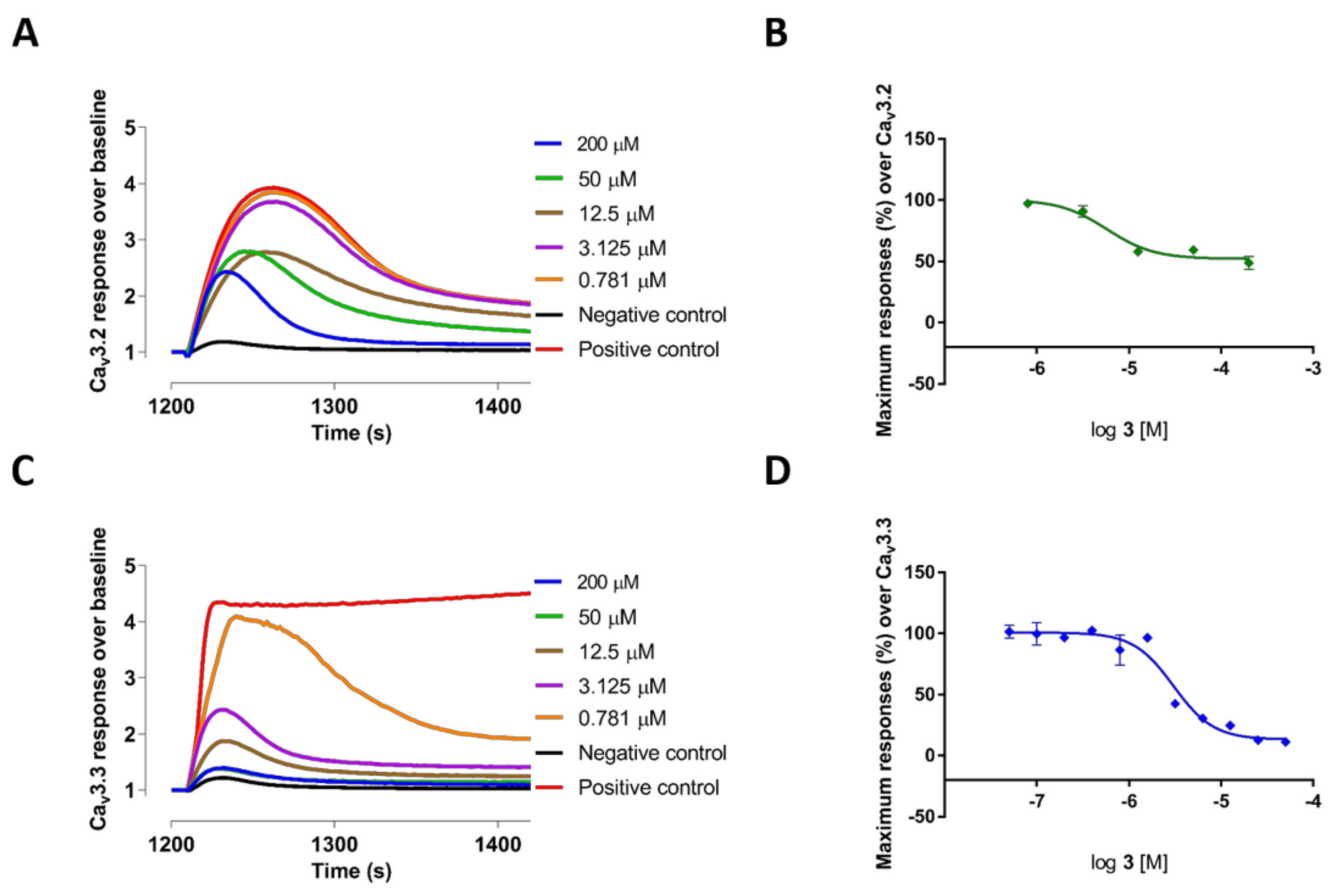
(**A**) Representative fluorescent traces of the Ca_V_3.2 window current before and after addition of compound **3**; (**B**) the concentration-response curve for compound **3** (IC_50_ = 5.77 μM, *n =* 4); (**C**) representative fluorescent traces of the Ca_V_3.3 window current before and after addition of compound **3**; and (**D**) the concentration response curve for compound **3** (IC_50_ value of 3.05 μM, *n =* 4).

The 2,2-bis (3,3′-indolyl) propionic acid **4** and the bisindole alkaloid **3a** showed poor inhibition of all the Ca^2+^ responses. In contrast, methyl-l-tryptophan-analog **3** showed good potency and selectivity for Ca_V_3.3 currents measured in T-type window current assays with an IC_50_ value of 3.81 ± 1.08 µM (*n =* 3), which was >8-fold better than its potency at high voltage-activated (HVA) Ca_V_s: **3** also potently blocked Ca_V_3.2 responses with an IC_50_ value of 6.59 ± 0.66 µM (*n =* 3). Comparatively, deoxy pseudellone C (**2**), which had tryptophan moiety stabilized with a methyl amide group, had a 2-fold reduced potency for Ca_V_3.3 window currents with an IC_50_ value of 7.71 ± 0.23 µM (*n =* 3) and a ~3-fold reduced potency for Ca_V_3.2 window currents with an IC_50_ value of 18.24 ± 0.49 µM (*n =* 3), compared to **3**. The natural product pseudellone C (**1**), which was generated by oxidation of the methylene group of the tryptophan moiety of deoxy pseudellone C (**2**), had >3-fold further reduced potency for Ca_V_3.3 compared to **2**. The fluorescent Ca^2+^ responses before and after addition of compounds **2** and **3**, and their representative concentration response curves, are presented in Figure 1 (**2**) and Figure 2 (**3**), respectively.

**Figure 3:**
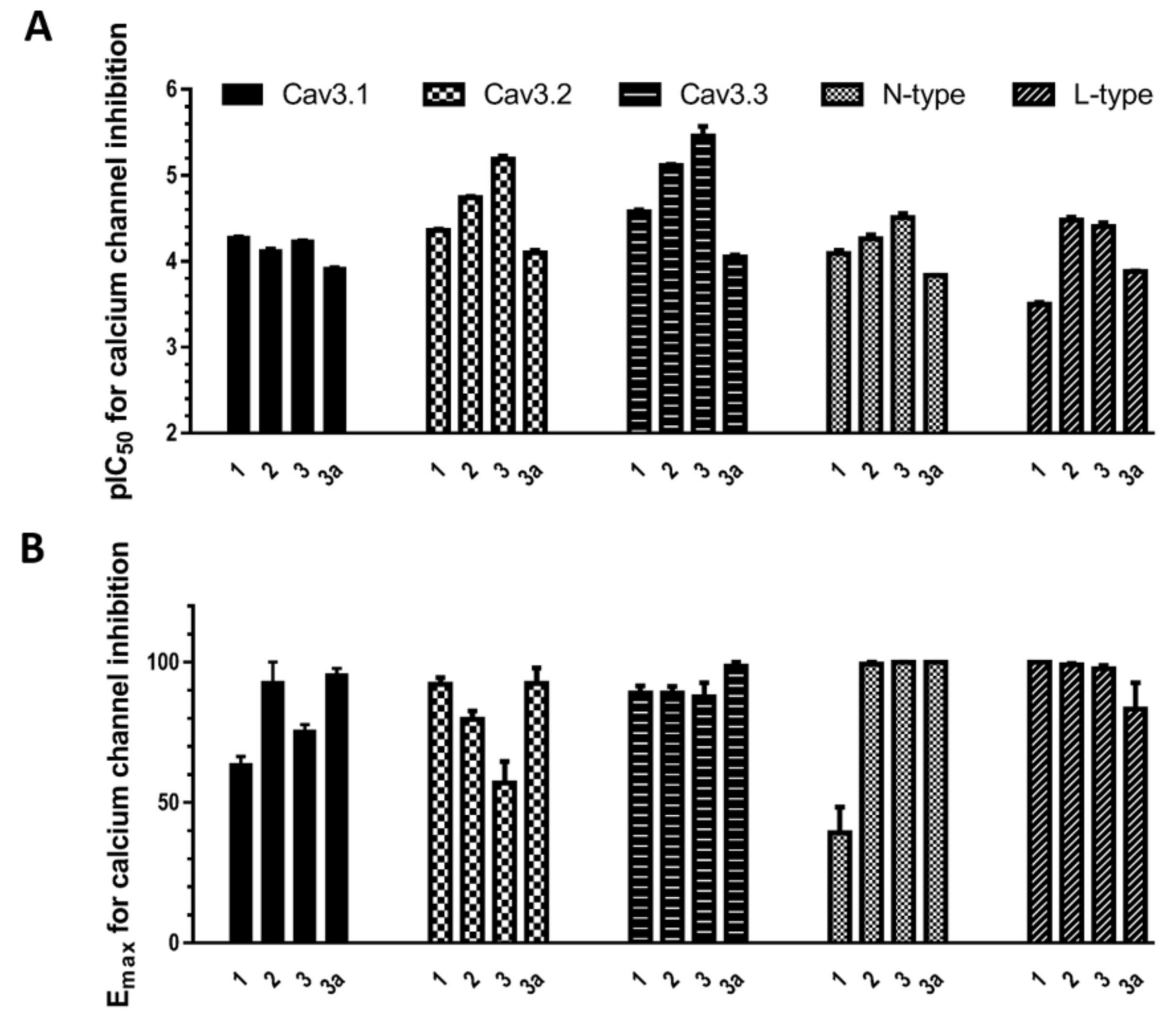
(**A**) pIC_50_ values for bisindole alkaloids **1**, **2**, **3**, and **3a** inhibition of T-, N- and L-type calcium channels; (**B**) *E_max_* values for bisindole alkaloids **1**, **2**, **3**, and **3a** inhibition of calcium channels. Here, **1** produced 63.56% ± 2.80% (*n =* 3) inhibition for Ca_V_3.1 and 39.26% ± 9.18% (*n =* 3) inhibition for N-type Ca_V_2.2, and **3** produced 75.47% ± 2.29% (*n =* 3) inhibition for Ca_V_3.1 and 56.91% ± 7.74% (*n =* 3) inhibition for Ca_V_3.2.

Interestingly, as indicated in Figure 2A,B and Figure 3, compound **3** showed potent partial blocking of the Ca_V_3.2 window current. Also, pseudellone C is a weak calcium channel blocker that showed partial blocking of Ca_V_3.1 and Ca_V_2.2. Partial blocking of Ca_V_3.x has been indicated to be useful for the treatment of epileptic behaviors by reducing synchronized oscillations of thalamocortical neurons [13,14]. Currently, compound **3** analogs are being designed for potential in rodent models of absence epilepsy and pain.

### 2.3. Electrophysiological Characterization of the Selective Ca_V_3.x Blockers in QPatch Assays

**Figure 4:**
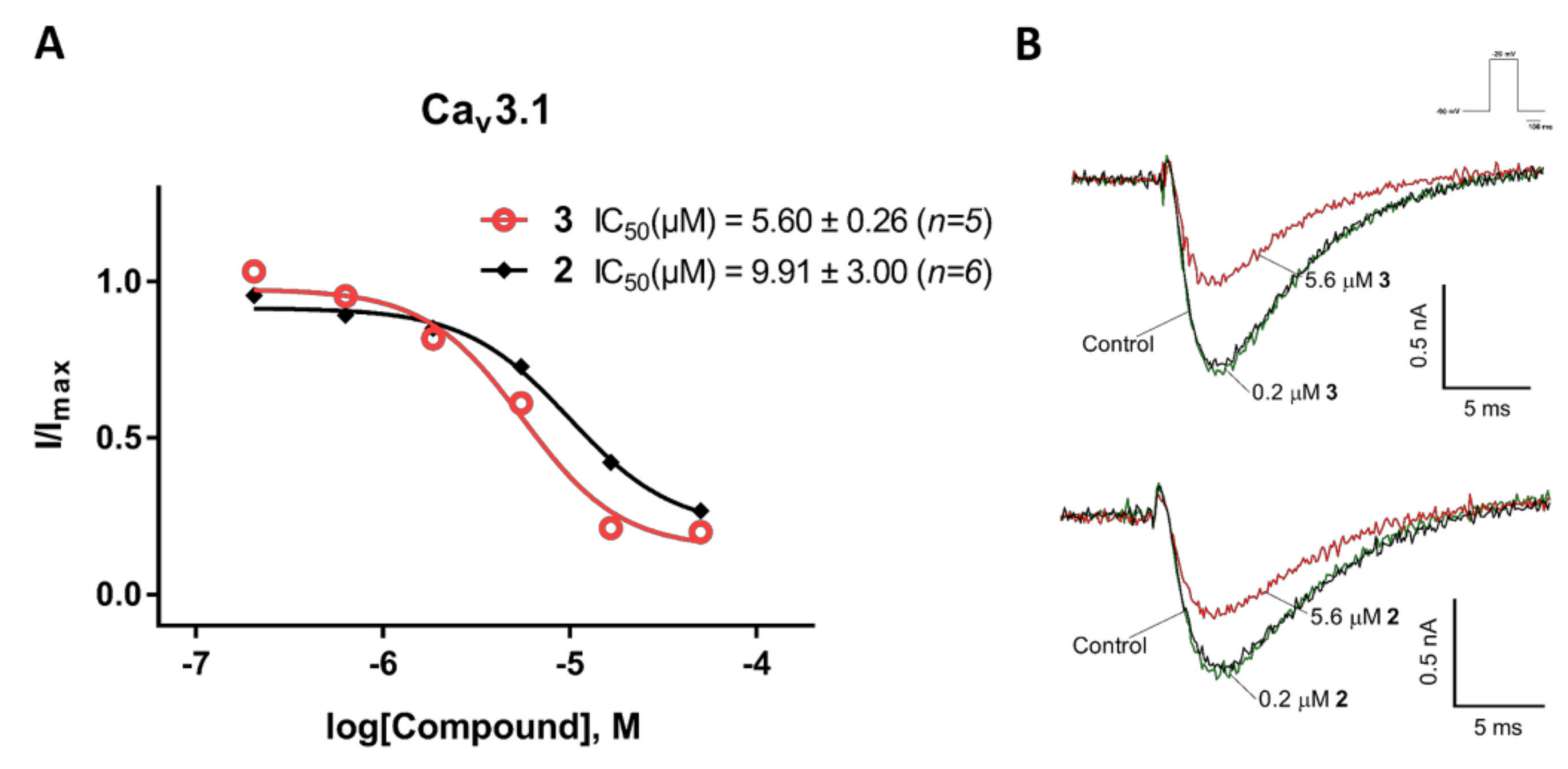
Inhibition of Ca_V_3.1 current by compounds **2** and **3**. (**A**) Concentration-response curves of compounds **2** and **3** on recombinant hCa_V_3.1 channels (*n =* 5–6). Data are means ± SEM. (**B**) Representative *I*_Ca_ during 200 ms depolarizations to *V*_max_ (−20 mV) from a holding potential of −90 mV before and after perfusions of 0.2 µM and 5.6 µM of compounds **2** and **3,** as indicated.

**Figure 5:**
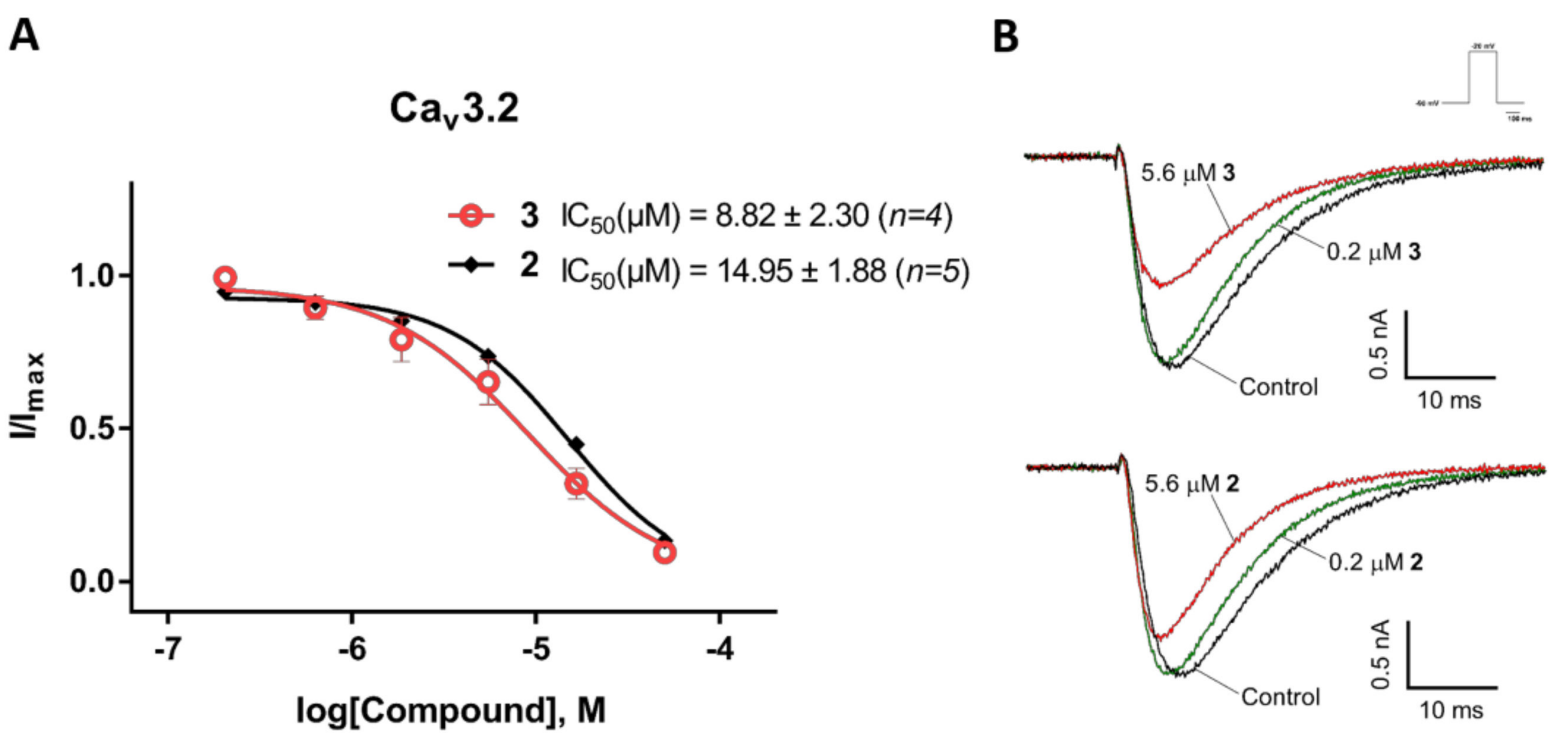
Inhibition of Ca_V_3.2 current by compounds **2** and **3**. (**A**) Concentration-response curves of compounds **2** and **3** on recombinant hCa_V_3.2 channels (*n =* 4–5). Data are means ± SEM. (**B**) Representative *I*_Ca_ during 200 ms depolarizations to *V*_max_ (−20 mV) from a holding potential of −90 mV before and after perfusions of 0.2 µM and 5.6 µM of compounds **2** and **3,** as indicated.

**Figure 6:**
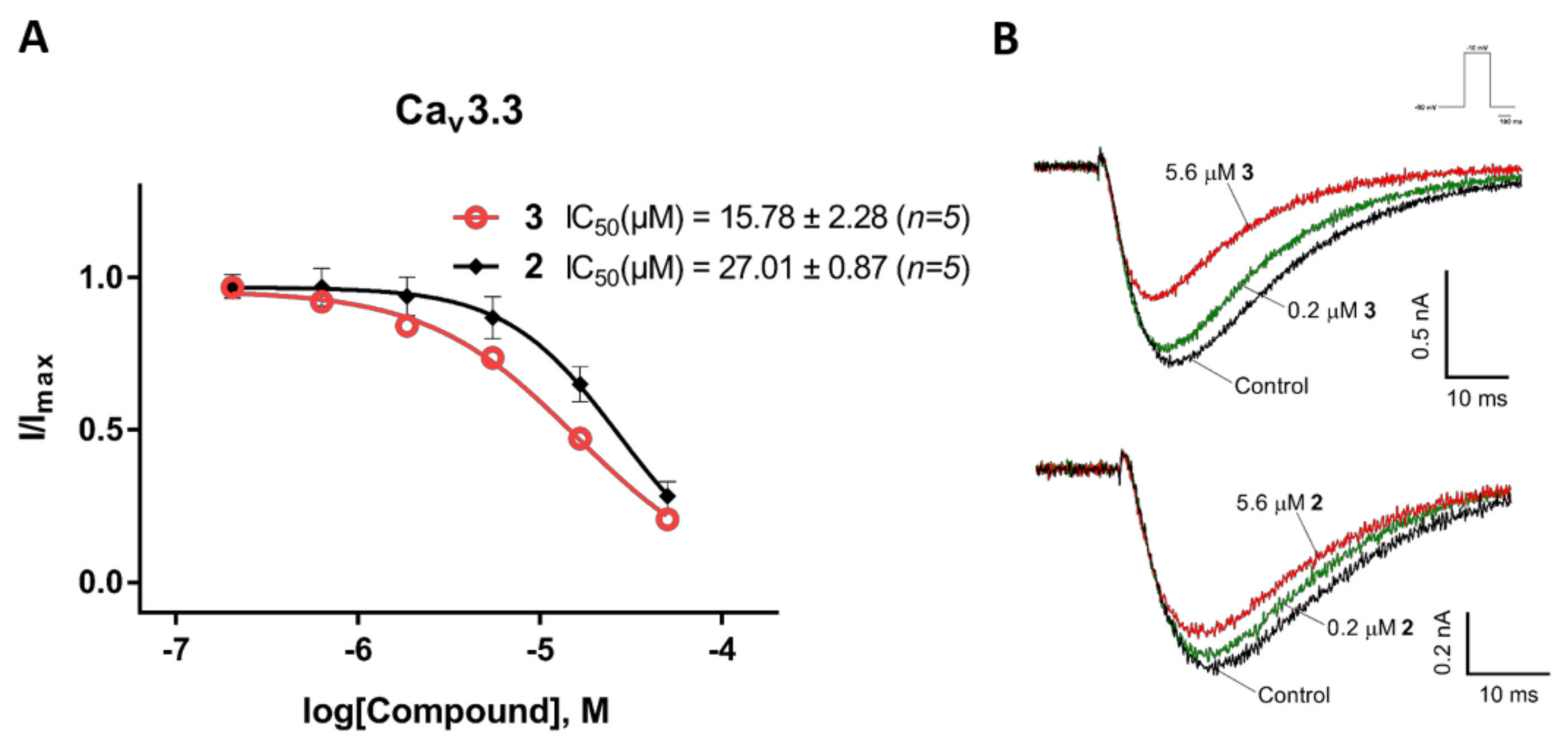
Inhibition of Ca_V_3.3 current by compounds **2** and **3**. (**A**) Concentration-response curves of compounds **2** and **3** on recombinant hCa_V_3.3 channels (*n =* 5). Data are means ± SEM. (**B**) Representative *I*_Ca_ during 200 ms depolarizations to *V*_max_ (−10 mV) from a holding potential of −90 mV before and after perfusions of 0.2 µM and 5.6 µM of compounds **2** and **3,** as indicated.

We also examined the effects of compounds **2** and **3** on the Ca_V_3.x by whole-cell patch-clamp using the automated electrophysiology platform QPatch 16 X (Figure 4, Figure 5 and Figure 6). Surprisingly, both compounds showed preferential blocking of Ca_V_3.1 currents (**2**: IC_50_ = 5.60 ± 0.26 µM (*n =* 5); **3**: IC_50_ = 9.91 ± 3.00 µM (*n =* 6)). Unlike the full inhibition by **2** and **3** of Ca_V_3.2 and Ca_V_3.3 whole-cell currents (*E_max_* value ≥ 95), **2** and **3** only showed partial block of the Ca_V_3.1 current, with an *E_max_* value of 70.31 ± 2.13 (*n =* 6) and 82.84 ± 2.55 (*n =* 5), respectively. Although **2** and **3** did not show comparably good potency for the Ca_V_3.3 peak current compared to their effects on the Ca_V_3.3 window current, low concentrations (200 nM) of **2** and **3** both showed weak inhibition (<10%) of the Ca_V_3.3 peak current.

**Figure 7:**
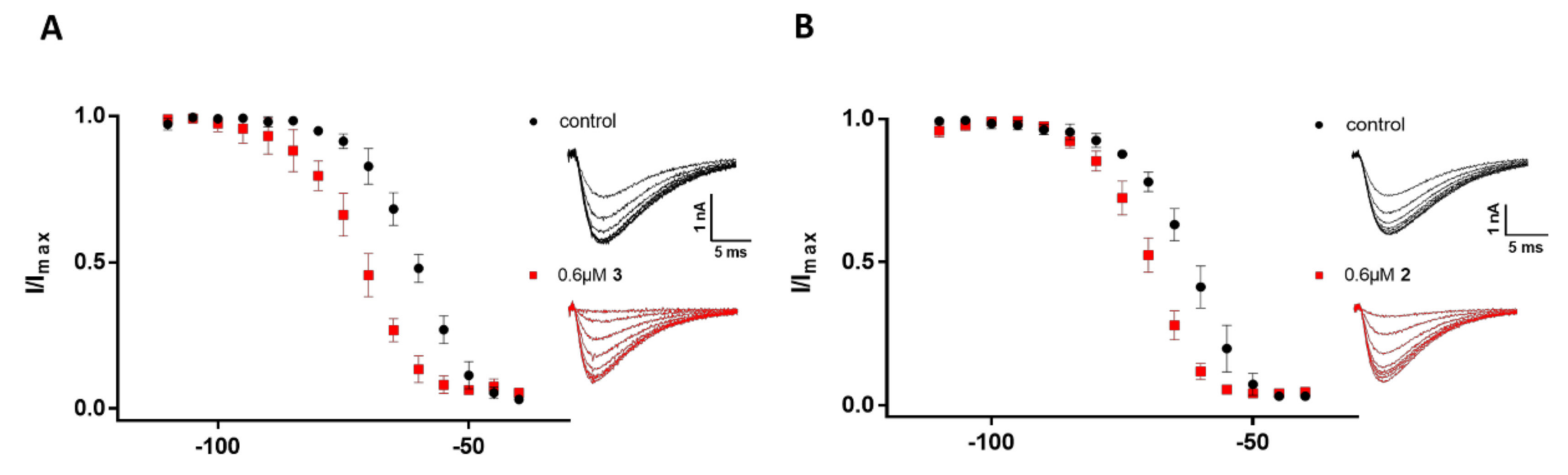
Comparison of the steady-state inactivation kinetics of Ca_V_3.2 before (black) and after (red) the addition of 0.6 µM of **2** and **3**. Steps lasting 1 s were given directly from −110 mV to the indicated voltages in 5-mV increments, followed by 60-ms test pulses to −20 mV. (**A**) Normalized *I*/*I*_max_ under conditioning steps from −110 mV to −40 mV (*n =* 5), along with representative elicited *I*_Ca_ under potentials from −95 mV to −60 mV before (black) and after (red) the addition of 0.6 µM of **3**. Error bars represent the SEM. (**B**) Normalized *I*/*I*_max_ under conditioning steps from −110 mV to −40 mV (*n =* 4), along with representative elicited *I*_Ca_ under potentials from −95 mV to −60 mV before (black) and after (red) the addition of 0.6 µM of **2**. Error bars represent the SEM.

It is noteworthy that both **2** and **3** had a significant voltage-dependent effect on steady-state inactivation of Ca_V_3.2, shifting half-maximal inactivation *V*_50_ to hyperpolarized potentials by ~6 mV and 11 mV, respectively (Figure 7). On average, control *V*_50_ (−65.40 ± 1.91 mV, *n =* 9) was shifted to −76.36 ± 1.56 mV (*n =* 5, *p* < 0.01) in the presence of compound **3**, whereas in the presence of **2** the *V*_50_ was shifted to −72.40 ± 1.83 mV (*n =* 4, *p* < 0.05). In contrast, the voltage-dependence of steady-state inactivation for Ca_V_3.1 and Ca_V_3.3 was not significantly shifted in the presence of **2** and **3**.

## 3. Discussion

In this study, we demonstrated that two pseudellone C-derived bisindole alkaloids, **2** and **3**, are potent and selective Ca_V_3.x current blockers that showed preferential inhibition of Ca_V_3.3 window currents measured in FLIPR assays and Ca_V_3.1 whole-cell currents measured in QPatch assays. The FLIPR window current assay applied to Ca_V_3.x was permissive of a window current resulting from incomplete inactivation [15]. The specific Ca_V_3.x window current made Ca_V_3.x a privileged gate for the entry of extracellular Ca^2+^ during secretion, neurotransmission, and cell proliferation [16,17]. Therefore, the inhibition of window currents in Ca_V_3.x should now be targeted for the development of analgesic, antiepileptic, and anticancer drugs. Novel Ca_V_3.x blockers that showed prominent experimental analgesic and antiepileptic efficacy [14,15,18] such as TTA-A2, Z941, and Z944, all showed better potency for T-type window currents than the activated current generated from the channel-closed state [14,15].

As mentioned above, tryptophan is the precursor for a wide range of pharmacologically important indole alkaloids [11,12]. Tryptophan-derived indole alkaloids have been receiving wide attention in drug discovery for their promising therapeutic potentials, represented by the powerful chemotherapy drugs vinblastine and vincristine [19,20,21] and the antipsychotic and antihypertensive drug reserpine [22,23,24]. In short, the marine fungal natural product pseudellone C was produced via oxidation of the intermediate **2**, which in turn was obtained from amidation of the carboxylic acid **3a**. The acid **3a** was generated from hydrolysis of **3**, which was in turn obtained from amide coupling of **4** and l-tryptophan methyl ester. The resulting suite of compounds have been evaluated for activity on VGCCs using FLIPR cell-based assays, and **2** and **3** were further explored for effects on Ca_V_3.x whole-cell currents in QPatch assays.

Both compounds **2** and **3** inhibited Ca_V_3.3 currents in the FLIPR window current assay, but to our surprise, they did not show comparable potency for Ca_V_3.3 whole-cell currents in QPatch assays, where affinity is determined by interactions with the resting state of the channel. In contrast, **2** and **3** both showed >7-fold better potency for Ca_V_3.1-activated currents than Ca_V_3.1 window currents, whereas for Ca_V_3.2, these two compounds did not show any preference for Ca_V_3.2 window currents or Ca_V_3.2-activated currents. Moreover, by measuring the voltage dependence of steady-state inactivation of Ca_V_3.x, the addition of both **2** and **3** significantly shifted the half-maximal inactivation *V*_50_ of Ca_V_3.2 to more hyperpolarized potentials, whereas they did not significantly alter Ca_V_3.1 and Ca_V_3.3 channel inactivation. In brief, these two new Ca_V_3.x blockers preferentially blocked either the window or activated current depending on the subtype and exclusively modulated channel inactivation kinetics for Ca_V_3.2, which may help in differentiating subtype-specific properties in further pharmacological studies on T-type Ca_V_3.x.

In summary, through a five-step total synthesis of marine fungal product pseudellone C, we achieved several bisindole alkaloid analogs of pseudellone C, two of which later revealed promising and selective inhibition of T-type Ca_V_3.x currents. For the first time, naturally sourced bisindole alkaloids were characterized as Ca_V_3.x blockers. Moreover, the exquisite differential inhibition of compounds **2** and **3** for the three T-type subtypes makes them useful pharmacological tools to probe the roles of individual Ca_V_3.x subtypes, and they could be subjected to further structural modification for the development of analgesic, antiepileptic, and anticancer drug candidates through targeting Ca_V_3.x.

## 4. Materials and Methods

### 4.1. General Procedure for Chemical Synthesis

All reagents were used as purchased from sigma Aldrich without further purification. Anhydrous solvents were used. Flash column chromatography was performed using ethylacetate (EtOAc) and petroleum ether as solvent. Preparative HPLC was used for the purification of the compounds using water and acetonitrile containing 0.01% TFA as solvent. ^1^H and ^13^C NMR spectra were recorded on Bruker (600 MHz) spectrometers. Data for ^1^H NMR spectra are reported as chemical shift (ppm), multiplicity (s = singlet, d = doublet, t = triplet, q = quartet, m = multiplet, dd = doublet of doublets, dt = doublet of triplets, br s = broad singlet), and coupling constant (*J* in Hz). Electrospray ionization mass spectrometry (ESIMS) experiments were carried out on a LC/MSD (quadrupole) instrument in both positive and negative modes. High-resolution ESIMS spectra were obtained on a micrOTOF mass spectrometer by direct injection in MeCN at 3 μL/min using sodium formate clusters as an internal calibrant.

#### 4.1.1. Synthesis of 2,2-di(1H-indol-3-yl)propanoic Acid (**4**)

To a solution of indole (2.34 g, 20 mmol) in anhydrous CH_3_CN (20 mL), we added pyruvic acid (1.32 g, 30 mmol, 1.5 equiv.) and FeCl_3_ (5 mol % 162 mg). The mixture was stirred under nitrogen at room temperature for 12 h until TLC revealed the disappearance of the starting material. The solvent was removed in vacuo, and the residue was purified by flash column chromatography (30% EtOAc/petroleum ether) to give **4** (2.58 g, 85%) as a reddish solid. ^1^H NMR (600 MHz, DMSO-*d*_6_): δ 10.85 (s, 2H), 7.33 (d, *J* = 8.09, 1H), 7.31 (d, *J* = 8.02, 1H), 7.03 (s, 1H), 7.02 (s, 1H), 7.00 (t, *J* = 8.09, 2H), 6.81 (t, *J* = 8.02, 2H), 1.97 (s, 3H). ^13^ C NMR (150 MHz, DMSO-*d*_6_): δ 176.1, 136.7, 125.9, 123.0, 120.7, 120.5, 118.1, 117.9, 111.4, 29.7, 25.9. HRMS (ESI) calcd for C_19_ H_16_ N_2_ O_2_ [M + Na]^+^ 327.1104 found *m/z* = 327.1096.

#### 4.1.2. Synthesis of l-Tryptophan Methyl Ester

To a solution of l-tryptophan (0.204 g, 1 mmol) in anhydrous MeOH (5 mL), we added thionyl chloride (0.236 g, 2 equiv.) at 0 °C in dropwise and stirred for 12 h at room temperature until TLC revealed the disappearance of the starting material. The solvent was removed in vacuo and the residue was purified by flash column chromatography (45% ethyl acetate in petroleum ether) to give **5** (0.163 g, 75%) as a white solid. ^1^H NMR (600 MHz, DMSO-*d*_6_): δ 11.14 (s, 3H), 8.66 (br s, 2H), 7.51 (d, *J* = 8.12, 1H), 7.36 (d, *J* = 8.12, 1H), 7.25 (s, 1H), 7.08 (t, *J* = 7.42, 1H), 7.00 (t, *J* = 7.19, 1H), 4.19 (t, *J* = 5.84, 1H), 3.63 (s, 3H) 3.36–3.27 (m, 2H). ^13^C NMR (150 MHz, DMSO-*d*_6_): δ 169.7, 136.1, 126.8, 124.9, 121.1, 118.6, 117.9, 111.5, 106.3, 52.6, 52.5, 26.0. HRMS (ESI) calcd for C_12_ H_15_ N_2_ O_2_ [M + H]^+^ 219.1128 found *m/z* = 219.1137.

#### 4.1.3. Synthesis of Methyl (2,2-di(1H-indol-3-yl)propanoyl)-l-tryptophanate (**3**)

A solution of **4** (0.304 g, 1 mmol) in DMF (3 mL) with DCC (200 mg, 1.5 mmol) and HOBT (135 mg, 1 mmol) was stirred at room temperature. After 15 min, L-methyl ester tryptophan (0.218 g, 1 mmol) and TEA (404 mg, 4 mmol) were added, and the reaction mixture was stirred at 60 °C for 1 h. After the completion of the reaction, ice-cold water was used to quench the reaction and the mixture was extracted with ethyl acetate (3 × 15). The extract was concentrated in vacuo, and the residue was purified via flash column chromatography (40% EtOAc in petroleum ether) to yield **3** as an off-white solid (38%). Here, [α]_D_^30^ = −21 (c = 0.01, methanol). ^1^H NMR (600 MHz, Acetone-*d*_6_, ppm): δ 10.11 (s, 1H), 10.06 (s, 1H), 9.76 (s, 1H), 7.41 (d, *J* = 8.15, 1H), 7.39 (d, *J* = 8.15, 1H), 7.32 (d, *J* = 8.15, 1H), 7.26 (d, *J* = 9.16, 1H), 7.26 (s, 1H), 7.24 (d, *J* = 8.03, 1H), 7.22 (d, *J* = 8.03, 1H), 7.05–7.00 (m, 4H), 6.93 (t, *J* = 7.06, 1H), 6.81 (t, *J* = 8.03, 1H), 6.79 (t, *J* = 7.13, 1H), 6.59 (d, *J* = 7.13, 1H), 6.10 (s, 1H), 4.77–4.74 (m, 1H), 3.57 (s, 3H), 3.10–2.96 (m, 2H), 2.85 (s, 3H). ^13^C NMR (150 MHz, Acetone-*d*_6_): δ 175.2, 173.0, 138.3, 137.4, 128.1, 127.1, 124.8, 124.6, 124.1, 122.1, 122.0, 121.9, 121.9, 121.5, 119.6, 119.5, 119.3, 118.8, 112.4, 112.3, 112.1, 109.8, 53.8, 52.1, 47.85, 28.0, 26.1. HRMS (ESI) calcd for C_31_ H_28_ N_4_ O_3_ [M + Na]^+^ 527.2054 found: *m/z* = 527.2054.

#### 4.1.4. Synthesis of (2,2-di(1H-indol-3-yl)propanoyl)-l-tryptophan (**3a**)

A solution of **3** (0.035 g, 0.07 mol) in 1:1 (MeOH/H_2_O) and LiOH (3.22 mg, 2 equiv.) was stirred for 3 h at pH 3 to yield a white solid that was filtered, dried, and purified by flash column chromatography to yield **3a** (29 mg, 85%) as a white solid. Here, [α]_D_^30^ = −14 (c = 0.01, methanol). ^1^H NMR (600 MHz, Acetone-*d*_6_): δ 10.13 (s, 1H), 10.07 (s, 1H), 9.77 (s, 1H), 7.41–7.38 (m, 2H), 7.33–7.30 (m, 2H), 7.29 (d, *J* = 2.48, 1H), 7.27 (d, *J* = 8.19, 1H), 7.20 (d, *J* = 8.04, 1H), 7.03–7.00 (m, 3H), 6.98 (d, *J* = 2.58, 1H), 6.92–6.90 (m, 1H), 6.82–6.79 (m, 1H), 6.77–6.74 (m, 1H), 6.62 (d, *J* = 7.05, 1H), 6.13 (d, *J* = 2.3, 1H), 4.70–4.74 (m, 1H), 3.12–3.03 (m, 2H), 2.06 (s, 3H). ^13^ C NMR (150 MHz, Acetone-*d*_6_): δ 175.5, 173.4, 138.3, 138.2, 137.4, 128.2, 127.1, 127.0, 124.8, 124.6, 124.1, 122.0, 121.98, 121.93, 121.8, 121.4, 119.7, 119.5, 119.4, 119.2, 119.0, 112.3, 112.4, 112.3, 112.0, 110.0, 53.6, 47.8, 28.0, 26.1. HRMS (ESI) calcd for C_30_ H_26_ N_4_ O_3_ [M + Na]^+^ 513.1897 found *m/z* = 513.1886.

#### 4.1.5. Synthesis of (S)-*N*-(3-(1H-indol-3-yl)-1-(methylamino)-1-oxopropan-2-yl)-2,2-di(1H-indol-3-yl)propanamide (**2**)

A solution of **3a** (0.15 g, 0.29 mmol) in DMF (3 mL) with DCC (200 mg, 1.5 mmol) and HOBT (135 mg, 1 mmol) was stirred at room temperature. After 15 min methyl amine (3 equiv., ethanolic solution) and TEA (404 mg, 4 mmol) was added, and the reaction mixture was stirred at 60 °C for 1 h. After the completion of the reaction, cold water was used to quench the reaction and the mixture was extracted with EtoAc (3 × 15 mL), and the combined EtOAc concentrated in vacuo with the residue purified by HPLC (phenomenex Luna C_18_ 250 × 21 mm, 10 µm, 20 mL/min elution, 10% to 100% MeCN/H_2_ O with 0.01% TFA as modifier) yielded **2** (54 mg, 37%) as a white solid. Here, [α]_D_^30^ = 0.0 (c = 0.10, methanol) and [α]_D_^30^ = 0.0 (c = 0.10, methanol). ^1^H NMR (600 MHz, Acetone-*d*_6_): δ 10.17 (s, 1H), 10.07 (s, 1H), 9.89 (s, 1H), 7.45 (d, *J* = 8.19, 1H), 7.39 (d, *J* = 8.21, 1H), 7.36–7.33 (m, 2H), 7.19 (d, *J* = 2.45, 1H), 7.10–7.05 (m, 3H), 7.01 (t, *J* = 7.60, 1H), 6.93 (t, *J* = 7.60, 1H), 6.89–6.88 (m, 2H), 6.72 (d, *J* = 7.44, 1H), 6.68 (t, *J* = 7.45, 1H), 6.34 (s, 1H), 6.26–6.19 (m, 1H), 4.68 (q, *J* = 6.52, 1H), 3.33 (s, 1H), 3.13–2.96 (m, 2H) 2.31 (d, *J* = 4.50, 3H), 2.04 (s, 3H). ^13^C NMR (150, Acetone-*d*_6_): δ 175.4, 172.5, 138.3, 137.5, 128.3, 126.9, 126.6, 124.9, 124.3, 121.1, 122.0, 121.9, 121.5, 119.53, 119.52, 119.4, 119.2, 112.6, 112.5, 112.0, 54.7, 47.8, 28.5, 26.1, 25.9. HRMS (ESI) calcd for C_31_ H_29_ N_5_ O_2_ [M + Na]^+^ 526.2213 found *m/z* = 526.2228.

#### 4.1.6. Synthesis of Pseudellone C (**1**)

A solution of **2** (35 mg, 0.07 mol) in 9:1 MeCN/H_2_O (1 mL) was treated with DDQ (32 mg, 0.14 mmol) in 6 portions over intervals of 10 min, and the resulting mixture was stirred at room temperature for 3 h, after which the residue was dissolved in EtOAc, washed with saturated NaCl (5 mL) and water (10 mL), dried over anhydrous Na_2_ SO_4_, and concentrated in vacuo to give residue, which was purified by HPLC (phenomenex Luna C_18_ 250 × 21 mm, 10 µm, 20 mL/min elution, 10% to 100% MeCN/H_2_ O with 0.01% TFA as modifier) to afford pseudellone C (14.4 mg, 97%) as a white solid. Here, [α]_D_^30^ = 0.0 (c = 0.10, methanol) and ^1^H NMR (600 MHz, Acetone-*d*_6_): δ 11.17 (s, 1H), 10.25 (s, 1H), 10.21 (s, 1H), 8.49 (d, *J* = 3.24, 1H), 8.14 (d, *J* = 7.44, 1H), 7.59, (d, *J* = 6.07, 1H), 7.50 (dt, *J* = 1.03, 8.07, 1H), 7.47 (m, 2H), 7.41 (d, *J* = 8.15, 1H), 7.38 (d, *J* = 8.14, 1H), 7.29 (d, *J* = 2.48, 1H), 7.26 (d, *J* = 2.47, 1H), 7.23–7.20 (m, 1H), 7.19–7.15 (m, 1H), 7.10–7.07 (m, 1H), 7.05–7.01, (m, 1H), 6.96–6.91 (m, 1H), 6.83–6.81 (m, 1H), 6.45 (d, *J* = 5.09, 1H), 5.69 (d, *J* = 5.97, 1H), 2.45 (d, *J* = 4.87, 3H), 2.14 (s, 3H). ^13^C NMR (150 MHz, Acetone-*d*_6_): δ 187.0, 175.4, 168.0, 138.4, 138.3, 137.6, 136.7, 127.0, 126.9, 126.8, 125.1, 124.9, 124.5, 124.3, 124.2, 123.1, 122.6, 122.2, 122.1, 121.6, 121.4, 119.7, 119.6, 119.5, 112.9, 112.7, 112.5, 62.4, 47.9, 26.2, 26.1. HRMS (ESI) calcd for C_31_ H_27_ N_5_ O_3_ [M + Na]^+^ 540.2006 found *m/z* = 540.2026.

### 4.2. Cell Culture and Transient Expression

The human embryonic kidney 293 (HEK 293) cell lines (from Emmanuel Bourinet, Montpellier, France) stably expressing Ca_V_3.2 or Ca_V_3.3 were cultured under 5% carbon dioxide at 37 °C in Dulbecco’s Modified Eagle Medium (DMEM) Glutamax (Gibco, Life Technologies, Carlsbad, CA, USA) supplemented with 10% (*v/v*) fetal bovine serum (FBS), 100 U/mL penicillin, 100 μg/mL streptomycin (Gibco, Life Technologies), and 750 μg/mL geneticin (G418) (Gibco, Life Technologies). The Chinese Hamster Ovary (CHO) cell lines (Emmanuel Bourinet, Montpellier, France) expressing Ca_V_3.1 were cultured under 5% carbon dioxide at 37 °C in Alpha Minimum Essential Media (MEM) Glutamax (Gibco, Life Technologies), supplemented with 10% (*v/v*) fetal bovine serum (FBS) and 300 μg/mL geneticin (G418) (Gibco, Life Technologies). The human neuroblastoma SH-SY5 Y cells (Victor Diaz, Goettingen, Germany) were cultured under 5% carbon dioxide at 37 °C in RPMI 1640 antibiotic-free medium (Invitrogen, Carlsbad, CA, USA), supplemented with 15% FBS and 2 mM GlutaMAX™ (Invitrogen). D-PBS (Gibco, Life Technologies) was used to wash the cells, and 0.25% Trypsin-EDTA (Gibco, Life Technologies) was used to detach cells from the flask surface. They were split in a ratio of 1:5 (ideally 1000 cells/cm^2^) when they reached 70%–80% confluence (every 2–3 days). Transiently transfected Ca_V_3.1HEK293 T cells were used in FLIPR Assays. HEK 293 T cells were cultured under 5% carbon dioxide at 37 °C in DMEM supplemented with 10% (*v/v*) FBS. D-PBS was used to wash the cells, and 0.25% Trypsin-EDTA was used to detach cells from the flask surface. The cells were split and seeded at 6 million cells per T175 flask, to reach 70%–80% confluence after 24 h. The next day, 12 μg DNA of human Ca_V_3.1 was incubated in 900 μL serum-free DMEM with 36 μL FuGENE HD transfection reagent (Promega Corporation, Madison, WI, US) (1:3 DNA/Fugene ratio) for 20 min, and then the mixture was added into the cell flask slowly, drop by drop. After the transfection, the cells were cultured under 5% carbon dioxide at 37 °C for 24 h and then moved to a 28 °C incubator.

### 4.3. T-Type Calcium Channel Window Current FLIPR Assays

HEK 293 cells stably expressing Ca_V_3.2 or Ca_V_3.3 were seeded into 384-well black wall clear bottom plates (Corning, Lowell, MA, USA) at a density of 30,000 cells per well. Transiently transfected Ca_V_3.1HEK293 T cells were seeded into 384-well black wall clear bottom plates at a density of 60,000 cells per well. Once the cells reached 90%–95% confluence after 24 h, the media were removed from the wells and replaced with 20 μL of 10% calcium 4 dye (Molecular Devices, Sunnyvale, CA, USA) in HBSS-HEPES (containing 5 mM KCl, 10 mM HEPES, 140 mM NaCl, 10 mM glucose, and 0.5 mM CaCl_2_, pH 7.4) with 0.1% bovine serum albumin (BSA). The cells were incubated for 30 min at 37 °C in the presence of 5% carbon dioxide. Each well on the reagent plates for the first addition contained 15 μL different concentrations of compounds dissolved in HBSS-HEPES containing 0.1% BSA and <0.1% DMSO and was incubated for 20 min after loading. Positive and negative controls contained 15 μL of HBSS-HEPES (0.1% BSA) alone. The plates were placed in the FLIPR^TETRA^ (Molecular Devices, Sunnyvale, CA, USA) programmed to measure maximum fluorescence intensity following a second addition of the agonist 5 mM CaCl_2_. The data acquisition parameters were adjusted as follows: Baseline fluorescence of 1500–2000 arbitrary fluorescence units (AFU), emission wavelength of 515–575 nm, and excitation wavelength of 470–495 nm. HBSS-HEPES (0.1% BSA) was used in the second addition as a negative control. One fluorescence reading was taken before the second addition. Then the fluorescence readings were recorded every two seconds for 300 s. Raw fluorescence readings in the form of relative light units were converted to response over baseline using ScreenWorks^®^ (Molecular Devices, version 3.2.0.14) software. Using this approach, pimozide (Ca_V_3.2, IC_50_ = 4.2 ± 1.06 μM (*n* = 3); Ca_V_3.3, IC_50_ = 7.71 ± 0.70 μM (*n* = 3)) and mibefradil (Ca_V_3.2, IC_50_ = 1.02 ± 0.14 μM (*n* = 3); Ca_V_3.3, 2.0 ± 0.2 μM (*n* = 3)) inhibited Ca_V_3.x calcium influx with IC_50_ s consistent with literature values [25].

### 4.4. HVA Calcium Channel FLIPR Assays

SH-SY5Y cells were seeded into 384-well black wall clear bottom plates at a density of 15,000 cells per well, resulting in 90%–95% confluence after 24 h. The media were then removed from the wells and replaced with 20 μL of 10% calcium 4 dye (Molecular Devices) in physiological salt solution (PSS) (containing 5.9 mM KCl, 1.4 mM MgCl_2_, 10 mM HEPES, 1.2 mM NaH_2_ PO_4_, 5 mM NaHCO_3_, 140 mM NaCl, 11.5 mM glucose, and 1.8 mM CaCl_2_, pH 7.4) with 0.1% BSA. As reported [26], for N-type calcium channel FLIPR assays the cells were pre-incubated with 10 µM nifedipine added in the dye to ensure full inhibition of L-type calcium responses. For L-type calcium channel FLIPR assays, the cells were pre-incubated with 1 µM CVID added in the dye to ensure full inhibition of N-type calcium responses. Positive control on the first reagent plate contained 15 μL of PSS (0.1% BSA), whereas PSS (0.1% BSA) containing 1 µM CVID and 10 µM nifedipine (final concentration) was used as a negative control. The fluorescence readings were recorded and converted as described previously [26], and agonist containing 90 mM KCl + 5 mM CaCl_2_ was used in the second addition.

### 4.5. Whole-Cell Patch-Clamp Electrophysiology

Whole-cell patch-clamp experiments were performed on an automated electrophysiology platform QPatch 16 X (Sophion Bioscience A/S, Ballerup, Denmark) in single-hole configuration using 16-channel planar patch chip QPlates (Sophion Bioscience A/S). The extracellular recording solution contained, in mM: TEACl 157, MgCl_2_ 0.5, CaCl_2_ 5, and HEPES 10; pH 7.4 adjusted with TEAOH; and osmolarity 320 mOsm. The intracellular pipette solution contained, in mM: CsF 140, EGTA 1, HEPES 10, and NaCl 10; pH 7.2 adjusted with CsOH; and osmolarity 325 mOsm. Compounds were diluted in extracellular recording solution with 0.1% BSA at the concentrations stated (DMSO ≤ 0.1%), and the effects of compounds were compared to the control (extracellular solution with 0.1% BSA) parameters within the same cell. Compounds’ incubation time varied from two (for the highest concentration) to five (for the lowest concentration) minutes by applying the voltage protocol 10–30 times at 10 s intervals to ensure steady-state inhibition was achieved. The effects of compounds were obtained using 200 ms voltage steps to peak potential from a holding potential of −90 mV. The steady-state inactivation kinetics of Ca_V_3.x currents were examined by applying steps lasting 1 s from −110 mV to the indicated voltages in 5-mV increments, followed by 60-ms test pulses to −20 mV. Data were fitted with a single Boltzmann distribution: *I*/*I*_max_ = {1 + exp[*V* − *V*_50_]/*k*}^−1^, where *V*_50_ is the half-availability voltage and *k* is the slope factor. Off-line data analysis was performed using QPatch Assay Software v5.6 (Sophion Bioscience A/S) and Excel 2013 (Microsoft Corporation, Redmond, WA, USA).

### 4.6. Data Analysis

Data were plotted and analyzed using GraphPad Prism v7.0 (GraphPad Software Inc., San Diego, CA, USA). A four-parameter logistic Hill equation with variable Hill coefficients was fitted to the data for concentration-response curves. Data are means ± SEM of *n* independent experiments. Statistical analysis was performed with a paired Student’s *t*-test with statistical significance at *p* < 0.05.

## Figures and Tables

**Table 1:** Effects of pseudellone C-based bisindole compounds on low voltage-activated (LVA) and high voltage-activated (HVA) calcium channels.

Compounds	LVA Ca_V_s (IC_50_ (µM) *N* = 3)			HVA Ca_V_s (IC_50_ (µM) *N* = 3)	
	Ca_V_3.1	Ca_V_3.2	Ca_V_3.3	Ca_V_2.2	Ca_V_1
**1**	53.49 ± 2.72	43.96 ± 1.60	26.59 ± 1.23	82.55 ± 7.60	322.00 ± 21.63
**2**	76.36 ± 5.39	18.24 ± 0.49	7.71 ± 0.23	55.29 ± 5.58	33.66 ± 2.62
**3**	58.57 ± 2.62	6.59 ± 0.66	3.81 ± 1.08	31.32 ± 3.40	39.76 ± 3.98
**3a**	122.23 ± 5.96	80.65 ± 6.21	90.05 ± 4.99	146.70 ± 1.72	132.03 ± 1.63
**4**	72.28 ± 0.41	132.17 ± 6.13	119.17 ± 8.66	179.30 ± 19.68	264.30 ± 11.89
